# New Insights into the Metabolism of the Flavanones Eriocitrin and Hesperidin: A Comparative Human Pharmacokinetic Study

**DOI:** 10.3390/antiox10030435

**Published:** 2021-03-11

**Authors:** María Ángeles Ávila-Gálvez, Juan Antonio Giménez-Bastida, Antonio González-Sarrías, Juan Carlos Espín

**Affiliations:** Laboratory of Food and Health, Research Group on Quality, Safety and Bioactivity of Plant Foods, Department Food Science and Technology, Campus de Espinardo, CEBAS-CSIC, P.O. Box 164, 30100 Murcia, Spain; mavila@cebas.csic.es (M.Á.Á.-G.); jgbastida@cebas.csic.es (J.A.G.-B.); agsarrias@cebas.csic.es (A.G.-S.)

**Keywords:** flavanones, citrus, hesperidin, eriocitrin, hesperetin metabolites, eriodictyol metabolites, pharmacokinetics, lemon, orange, gut microbiota

## Abstract

The intake of hesperidin-rich sources, mostly found in orange juice, can decrease cardiometabolic risk, potentially linked to the gut microbial phase-II hesperetin derivatives. However, the low hesperidin solubility hampers its bioavailability and microbial metabolism, yielding a high inter-individual variability (high vs. low-producers) that prevents consistent health-related evidence. Contrarily, the human metabolism of (lemon) eriocitrin is hardly known. We hypothesize that the higher solubility of (lemon) eriocitrin vs. (orange) hesperidin might yield more bioavailable metabolites than hesperidin. A randomized-crossover human pharmacokinetic study (*n* = 16) compared the bioavailability and metabolism of flavanones from lemon and orange extracts and postprandial changes in oxidative, inflammatory, and metabolic markers after a high-fat-high-sugars meal. A total of 17 phase-II flavanone-derived metabolites were identified. No significant biomarker changes were observed. Plasma and urinary concentrations of all metabolites, including hesperetin metabolites, were higher after lemon extract intake. Total plasma metabolites showed significantly mean lower T_max_ (6.0 ± 0.4 vs. 8.0 ± 0.5 h) and higher C_max_ and AUC values after lemon extract intake. We provide new insights on hesperetin-eriodictyol interconversion and naringenin formation from hesperidin in humans. Our results suggest that regular consumption of a soluble and eco-friendly eriocitrin-rich lemon extract could provide a circulating concentration metabolites threshold to exert health benefits, even in the so-called low-producers.

## 1. Introduction

Epidemiological studies and meta-analyses have associated the intake of flavonoid-rich citrus fruit consumption with lower stroke risk [1] and the incidence of some types of cancer [2,3]. Hesperidin and eriocitrin are the main flavonoids (flavanones) present in citrus, such as orange and lemon, respectively. In contrast to other flavonoids widely present in plant foods, flavanones are present in our diet almost exclusively in citrus fruits and, to a lesser extent, in some aromatic herbs or tomatoes [4]. There is abundant evidence that reports the bioavailability, metabolism, and health-beneficial effects of hesperidin after hesperidin-rich sources intake in both animals and humans [5,6,7,8,9]. However, human studies on the bioavailability and metabolism of eriocitrin are much less abundant.

There is evidence supporting that citrus flavonoids may represent promising bioactive agents for treating metabolic and cardiovascular diseases [10,11]. In this regard, hesperidin is abundant in the Western diet, mainly through orange juice intake. Moreover, purified hesperidin is commercially available at low cost since it can be easily synthesized or extracted and highly purified industrially. However, the presence of eriocitrin, mainly occurring in lemons, in the Western diet is low, and the commercial availability of eriocitrin and eriocitrin-rich sources is very limited.

There are drugs like Daflon^®^ 500 mg (micronized purified flavonoid fraction of 50 mg flavonoids (expressed as hesperidin) plus 450 mg of diosmin, a semisynthetic analog) that are used in the treatment of venous insufficiency and has shown its effectivity in improving leg symptoms, edema, and quality of life in patients with the chronic venous disease [12,13]. Paradoxically, despite its medical prescription and commercialization, the European Food Safety Authority (EFSA) has refused the health claim “maintenance of physiological venous-capillary permeability” associated with the intake of flavonoids like hesperidin, diosmin, and troxerutin [14]. This rejection was based on the impossibility of establishing a cause-effect relationship between the consumption of those flavanones and the maintenance of normal venous-capillary permeability [14]. Overall, the substantial variability of results described in the scientific literature was crucial for the rejection of this health claim. The high variability might be due to: (i) the different subjects’ responses to polyphenols’ consumption [15,16,17], including flavanones like hesperidin [18], and (ii) the low bioavailability of some flavanones that could prevent some metabolites from not reaching a sufficient circulating concentration threshold to exert health benefits.

The health-related effects described after the intake of hesperidin-rich sources have been mostly attributed to hesperetin metabolites. Hesperidin has been reported to be resistant to degradation in the stomach and small intestine, in vitro and in vivo, but deconjugated on reaching the proximal colon [19,20,21]. Hesperetin is produced upon release of the rutinose moiety by the gut microbial rhamnosidases and further phase-II conjugations to yield circulating metabolites with anti-inflammatory and cardioprotective effects in preclinical studies [21,22,23,24]. However, the low hesperidin solubility hampers its bioavailability and microbial metabolism, yielding a high inter-individual variability in the production of hesperetin derivatives [18], which could prevent consistent health-related evidence [25,26]. Indeed, approaches such as the micronization and encapsulation of flavanones, including hesperidin, could increase the gut microbiota metabolism and production of hesperetin-derived metabolites [27]. Another approach to investigate the impact of solubility in flavanone metabolism is the comparison of hesperidin vs. eriocitrin. The high insolubility of hesperidin is mainly due to the presence of a methoxy (–OCH_3_) group in the 4′ position of its B-ring. However, eriocitrin contains two hydroxyl groups (–OH) groups in the 3′ and 4′ positions of its B-ring. The presence of this *ortho*-dihydroxyl moiety confers higher solubility and antioxidant activity to eriocitrin vs. hesperidin [28].

In this context, we hypothesize that the higher solubility of eriocitrin vs. hesperidin could facilitate the metabolism of eriocitrin to yield phase-II derived metabolites in a shorter time, including eriodictyol-derived metabolites, with increased antioxidant activity. With this in mind, we aimed at comparing the bioavailability and metabolism of hesperidin and eriocitrin in a pharmacokinetic study with healthy volunteers after consuming a hesperidin-rich orange extract and an eriocitrin-rich lemon extract. Secondarily, we examined possible post-prandial effects on different biochemical, metabolic, inflammatory, and oxidative markers after a high-fat-high-sugar meal.

## 2. Materials and Methods

### 2.1. Standards and Reagents

Organic solvents such as methanol (MeOH), acetonitrile (ACN), dimethyl sulfoxide (DMSO) were obtained from J.T. Baker (Deventer, The Netherlands). Milli-Q system (Millipore Corp., Billerica, MA, USA) ultra-pure water was used throughout this experiment. All chemicals and reagents were of analytical grade. The following chemicals were purchased from Sigma-Aldrich (St. Louis, MO, USA): butylhydroxytoluene (BHT); ethylenediaminetetraacetic acid (EDTA); apigenin (≥97%); hesperidin; hesperetin and rutin (≥95%). The flavanone standards eriocitrin, eriodictyol, homoeriodictyol, and narigenin were purchased from Extrasynthese (Genay, France). The metabolites hesperetin 7-*O*-glucuronide, hesperetin 3′-*O*-glucuronide, hesperetin 3-O-sulfate were obtained from Villapharma Research S.L. (Fuente Alamo, Murcia, Spain), and naringenin 4′-*O*-glucuronide and naringenin 7′-*O*-glucuronide from Bertin Pharma (Montigny le Bretonneux, France).

### 2.2. Analysis of Orange and Lemon Extracts by HPLC-DAD-MS/MS

Hesperidin-rich orange extract (CBCs, Citrus Bioflavonoids Complex, 15% standardized in hesperidin) was provided by Laboratorios Admira (Alcantarilla, Murcia, Spain). An aqueous-soluble eriocitrin-rich lemon extract (Wellemon™), obtained by an eco-friendly process, was provided by Euromed S.A. (Mollet del Vallès, Barcelona, Spain). The flavanone content of the extracts was analyzed to administer the same amount of both hesperidin and eriocitrin to the volunteers.

Fifteen mg of each citrus extract was weighed and extracted with 10 mL of DMSO [29]. Samples were vortexed for 1 min and centrifuged at 4000× *g* for 10 min at room temperature. The supernatant was collected, diluted in methanol, and filtered through a 0.45 µm polyvinylidene difluoride (PVDF) filter before analysis by HPLC-DAD-MS/MS. Three replicates were extracted and analyzed for each sample.

HPLC analyses were performed on an Agilent 1200 HPLC system with a photodiode array detector (DAD) (Agilent Technologies, Waldbronn, Germany) and an ion trap mass spectrometer detector in series (Bruker Daltonik, Bremen, Germany). A reverse-phase Pursuit XRs C18 column (250 × 4.0 mm, 5 µm) (Agilent Technologies) was used for the chromatographic separation with water:formic acid (99:1) (phase A) and acetonitrile (ACN) (phase B) as mobile phases at 0.8 mL/min and the following gradient: 0–10 min 1–15% B, 10–40 min, 15–40% B; 40–44 min, 40–90% B, 44–46 min 90% B. The initial conditions (1% solvent B) were recovered in 1 min and maintained for 6 min. The sample injection volume was 20 µL.

In the mass spectrometer, nitrogen was used as drying and nebulizing gas with pressure at 65 psi, flow 11 L/min, and temperature 350 °C. The capillary voltage was 4000 V. Mass (MS) spectra were recorded in negative mode with a full-scan acquisition in the *m*/*z* range 100–1100 with a target mass of 500 *m*/*z.* Auto-MS^n^ mode was applied to obtain information about the fragmentation patterns.

The UV-Vis chromatograms were acquired at 280, 320, 340, and 360 nm. Hesperidin and eriocitrin were quantified at 340 nm with their corresponding standards. The rest of the flavanones in the lemon extract were quantified using eriocitrin at 340 nm. Flavones were also quantified at 340 nm using the flavone apigenin, and flavonols at 360 nm, using rutin.

### 2.3. Volunteers and Study Design

The study protocol was conducted following the ethical recommendations of the Declaration of Helsinki and approved (reference 076/2019) by the Spanish National Research Council’s Bioethics Committee (Madrid, Spain).

The study was carried out in sixteen healthy subjects (8 men and 8 women) with average age and body mass index of 33.2 ± 7.2 years and 23.0 ± 2.1 kg/m^2^, respectively (Table 1). They were non-smoker, non-vegetarian, not taking any medication or nutritional supplements, and were not suffering from any chronic pathology or gastrointestinal disorder. The volunteers signed their informed consent before participation. The sample size was estimated attending to previous pharmacokinetic studies dealing with the bioavailability and metabolism of phenolic compounds [18,25,30]. Regarding the evaluation of post-prandial changes in different biomarkers, this was an exploratory study and we did not aim to evaluate specific changes in any variable.

The trial’s primary endpoint was to evaluate the time-course bioavailability and metabolism of hesperidin and eriocitrin in healthy human subjects using a pharmacokinetic approach. Secondary endpoints involved the evaluation of post-prandial changes in several biomarkers dealing with metabolism, inflammation, and oxidative stress after a high-fat-high-sugars meal. The design was a randomized pharmacokinetic, double-blind, crossover, single-dose trial consisted of 2 test-phases. The volunteers were randomly allocated into two groups using a computerized random number list. Each group consumed one of the citrus extracts by following a test-phase that lasted for 1 day for blood sampling and urine collection, a 2-week washout period between each test phase, and crossover before consuming the other extract. The volunteers were instructed to avoid the consumption of flavanone-containing products, from a list provided by the researchers, for 1 week before the study and during the wash-out phase (citrus fruit juices, tomatoes, tomato-based sauces (like ‘ketchup’), peppers, almonds, etc.).

**Figure 1:**
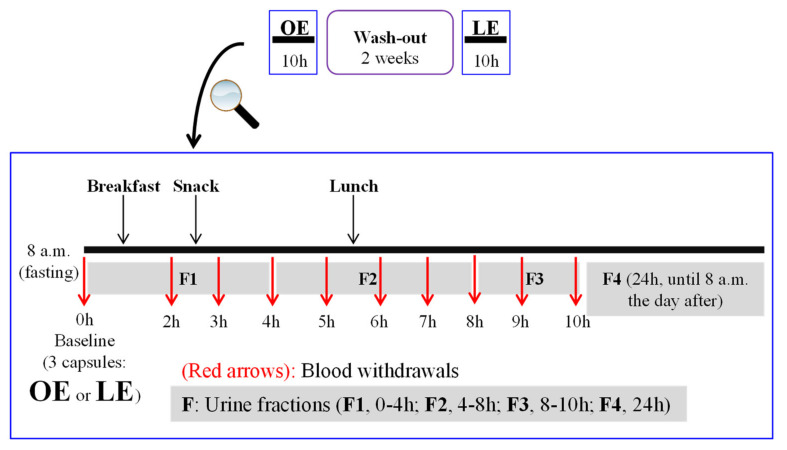
Study design. OE, orange extract; LE, lemon extract.

During each test-phase, fasting volunteers ingested 3 identical hard gelatine capsules at 8.00 a.m. The 3 capsules provided a total of 1.95 g orange extract, containing 260 mg hesperidin, and 3.1 g lemon extract, containing 260 mg eriocitrin. Blood samples were collected in EDTA-coated tubes at baseline (t = 0, before capsules intake) and 2, 3, 4, 5, 6, 7, 8, 9, and 10 h after consuming the extracts (Figure 1).

Plasma samples were obtained by centrifugation of peripheral venous blood at 14,000× *g* for 15 min at 4 °C and immediately frozen at −80 °C. Glucose, total cholesterol, HDL-cholesterol, LDL-cholesterol, triglycerides, insulin, homeostatic model assessment insulin resistance (HOMA-IR), gamma-glutamyl transferase (GGT), and alanine aminotransferase (ALT) were determined as previously reported [30,31].

Urine samples were collected at baseline, and after capsules intake, different urine fractions were collected, i.e., t = 0−4 h (F1); t = 4–8 h (F2), t = 8–10 h (F3), and t = 10–24 h (F4) (Figure 1). Untreated urine samples were aliquoted and frozen at −80 °C for up to 4 weeks until analyses, avoiding freeze-thaw cycles to avoid metabolites’ deconjugation. Moreover, three measurements of blood pressure and heart rate were taken before each time-point of blood withdrawal.

### 2.4. Diet

On each test phase, light and polyphenol-free breakfast and snack were provided 1 h and 2.5 h, respectively, after consumption of the capsules to prevent as much as possible potential interactions between food intake and polyphenols absorption. The volunteers consumed a high-fat-high-sugar meal for lunch (5.5 h after consuming the capsules to prevent any absorption interaction with the flavanones). The meal contained 1967 kcal (238 g of carbohydrates (83 g of free sugars), 73 g of proteins, and 92 g of fats), and consisted of a burger with bacon, chips, cheese sauce, and a piece of chocolate cake, plus 500 mL of coke^®^. Each volunteer drank between four and six glasses of water during the trial.

### 2.5. Analysis of Flavanone-Derived Metabolites in Urine and Plasma by UPLC-ESI-QTOF-MS

The analyses were performed on an Agilent 1290 Infinity UPLC system coupled to a 6550 Accurate-Mass quadrupole-time-of-flight (QTOF) mass spectrometer (Agilent Technologies, Waldbronn, Germany) using an electrospray interface (Jet Stream Technology). A previously validated method for analyzing phenolic metabolites in biological samples, including flavanone-derived metabolites, was used [29]. Briefly, plasma samples (300 µL) were extracted with 1 mL acetonitrile: formic acid (98:2, *v*/*v*). After centrifugation at 14,000× *g* for 10 min at 4 °C, the supernatant was evaporated in a speed vacuum concentrator. Finally, samples were re-suspended in 100 µL of methanol, filtered through a 0.22 µm cellulose filters before analysis by UPLC- ESI-QTOF-MS.

Urine samples were centrifuged at 14,000× *g* for 10 min and diluted (1/2; *v*/*v*) with acidified water (0.1% formic acid) and filtered through a 0.22 µm cellulose filters before analysis by UPLC-ESI-QTOF-MS.

A targeted screening strategy was applied to identify metabolites that could be present after consuming the citrus extracts [29]. Twenty-eight possible compounds were browsed in all the samples. The list of compounds included the polyphenols present in the extracts, with particular focus on the flavanone rutinosides hesperidin and eriocitrin, their respective aglycones hesperetin, and eriodictyol, as well as their derived phase-II conjugates (glucuronides, sulfates, sulfoglucuronides, etc.). The exact mass of each proposed compound was extracted using an extraction window of 0.01 *m*/*z*.

The metabolites hesperetin 7-*O*-glucuronide, hesperetin 3′-*O*-glucuronide, hesperetin 3-*O*-sulfate, naringenin 4′-*O*-glucuronide, and naringenin 7′-*O*-glucuronide were quantified with their corresponding standards. When no authentic standards were available, the identification of conjugated metabolites in plasma and urine was achieved by searching those metabolites, not present at baseline with the exact theoretical mass of the molecular ion, with a score higher than 90% and error lower than 5 ppm. The parent phenolic compounds were quantified with their corresponding standards. In the case of some relevant conjugated metabolites, which standards were not available (eriodictyol and homoeriodictyol conjugates), their quantification was achieved by using relative response factors (RRFs) as previously described [32]. Briefly, RRFs are useful to check the different responses of metabolites in the mass spectrometer vs. their UV signal. While the ionization response of free vs. conjugated forms is usually very different, their UV signal response is quite similar. To this purpose, we injected in a HPLC-DAD-ESI-Q (MS) the plasma samples (*n* = 5) with the highest concentrations of those conjugated metabolites. Concentrations were calculated by integrating the area from their corresponding UV peaks and compared with the UV peak area of a known concentration of their corresponding available parent compounds (eriodictyol and homoeriodictyol). The concentrations calculated were used to compare the MS response for each conjugated-aglycone pair.

### 2.6. Inflammation and Oxidative Markers in Urine and Plasma Samples

Biochemical markers of lipid-mediated post-prandial inflammation and some oxidative markers were evaluated as described elsewhere [33]. Briefly, plasma sample aliquots (300 µL) were thawed, and a 10 μL antioxidant solution (0.2 mg/mL BHT/EDTA in methanol/water (1/1)) was added. The extraction was performed using the solid-phase extraction cartridges Oasis HLB from Waters (Milford, MA, USA). Compounds were eluted using 2 mL of ethyl acetate. The rest of the protocol was the same above-mentioned used for the analysis of metabolites in plasma.

Thirty-eight metabolites (Supplementary Table S1), related to oxidative stress and inflammation that could be increased after the high-fat-high-sugar meal, were evaluated in urine and plasma. Most of the metabolites evaluated were oxylipins, which can derive from the degradation of fatty acids or different metabolic pathways related to inflammation involving the enzymes COX, LOX, and the CYP complex. Moreover, the oxidative stress-related metabolite 8-oxo-2′-deoxyguanosine was also included.

### 2.7. Statistical Analysis

Statistical analyses were carried out using the SPSS Software, version 23.0 (SPSS Inc., Chicago, IL, USA). Data are expressed as mean ± SD. The empirical distribution of data with the normality assumption was tested with the Shapiro-Wilk test. Depending on the normality test, either the parametric Student’s t-test or the non-parametric Mann-Withney-Wilcoxon test was used. Pharmacokinetic parameters were determined by non-compartmental analysis using the PKSolver software 2.0 (Microsoft Excel). Graphs and figures were performed using the Sigma Plot 13.0 (Systat Software, San Jose, CA, USA). For all the analyses, significant values were considered at *p* < 0.05.

## 3. Results

### 3.1. Analysis of Phenolic Compounds in the Citrus Extracts

Supplementary Figure S1 shows the UV chromatograms at 340 nm of orange and lemon extracts consumed by the volunteers. Peak numbers are detailed in Table 2, which summarizes the qualitative and quantitative results obtained by HPLC-DAD-MS/M. Remarkably, the “citrus bioflavonoids extract” was a 13.4% hesperidin product (133.7 ± 15.9 mg/g). No other phenolic was detected in this extract, and thus, “orange extract” refers to hesperidin from now on. The rest of the extract’s content is most likely maltodextrin, a known carrier in spray-dried extracts [34]. The main phenolic compound in the lemon extract was eriocitrin (83.3 ± 5.6 mg/g), with a concentration around 2.5-fold higher than another eriodictyol glycoside (compound 3, Table 1). The flavone C-glycosides represented 25% of the total content of the quantified phenolics in the lemon extract. Other quantified compounds included a flavonol (compound 12, Table 2) and a ferulic acid glucoside (compound 1, Table 2). Therefore, although the volunteers consumed an equal amount of hesperidin and eriocitrin (260 mg each), we recognize that the commercially available “orange extract” was only hesperidin (133.7 ± 15.9 mg/g), while lemon extract was a more complex mixture of citrus flavanones (240.16 ± 14.86 mg/g).

### 3.2. Post-Prandial Response after Consumption High-Fat-High-Sugar Meal

#### 3.2.1. Metabolic and Biochemical Markers

None of the baseline values of serobiochemical variables, blood pressure (diastolic and systolic), and heart showed differences in the two-phase test phases (Table 1). LDL-c values decreased, and heart rate, blood pressure, triglyceride, insulin, and HOMA-IR values significantly increased after consuming the high-fat-high-sugar meal vs. baseline regardless of the extract consumed (Supplementary Table S2). The rest of the markers did not significantly change upon consuming the meal.

#### 3.2.2. Inflammation and Oxidative Markers

None of the 38 metabolites browsed in urine samples and listed in Supplementary Table S1 were detected. However, seven plasma oxylipins were tentatively identified with an accurate mass, high score (>95%), and low error (<2 ppm) (Supplementary Table S3).

The oxylipins identified were hydroxyoctadecadienoic acid derivatives (HODEs), which are stable oxidation products from linoleic acid and alpha-linolenic acids. Depending on the oxylipin and(or) volunteer, the maximum values were detected between 1 and 3 h after the meal. Three oxylipins, i.e., 9,12,13-(TriHOME), 12(13)-DiHOME, and 9(10)-DiHOME), significantly increased after consuming the meal, regardless of the extract consumed.

### 3.3. Urinary Excretion of Phenolic Metabolites

**Figure 2:**
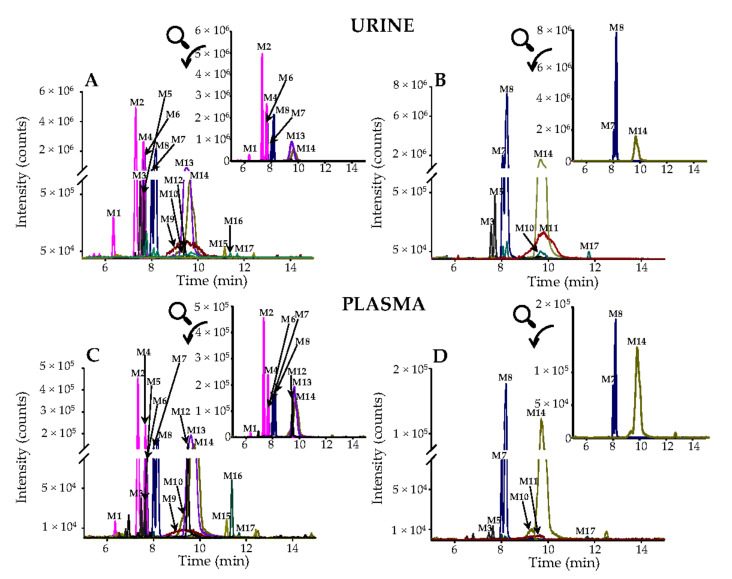
Representative extracted ion chromatograms (EICs) of urine and plasma metabolites after consuming (**A**,**C**) lemon or (**B**,**D**) orange extract. Numbers designate the metabolites according to Table 3.

Through a targeted metabolomic strategy, a total of 17 metabolites were tentatively identified (Table 3). No hesperidin or eriocitrin-derived metabolites were detected in any volunteer at baseline. All the identified metabolites were detected at least in one volunteer, independently of the extract consumed, except for the metabolites **M9** (eriodictyol sulfoglucuronide) and **M11** (hesperetin sulfoglucuronide), which only were detected after consuming lemon and orange extract, respectively (Table 3). The extracted ion chromatograms (EICs) of all the compounds detected are shown in Figure 2. These representative EICs illustrate the metabolites identified in urine (F3 fraction) after consuming lemon (Figure 2A) and orange (Figure 2B) extracts, and plasma after consuming lemon (Figure 2C) and orange (Figure 2D) extracts. From the 17 metabolites, 16 were detected after consuming lemon extract (except **M11**), and 15 after consuming orange extract (except **M9** and **M10** in urine, and **M9** and **M12** in plasma) (Table 3).

Table 4 shows the urinary excretion of quantified metabolites at the different fractions collected, i.e., 0–4 h (F1), 4–8 h (F2), 8–10 h (F3), and 10–24 h (F4), after extracts intake. The total excretion (24 h) of metabolites was significantly higher after consuming lemon extract than after consuming orange extract, considering the total amount of flavanones consumed.

### 3.4. Phenolic Metabolites in Plasma and Pharmacokinetic Evaluation.

All the metabolites quantified in urine were phase-II conjugates (glucuronides and sulfates), although the aglycone flavanones **M12**, **M15**, **M16**, and **M17** were also detected in some volunteers, mainly after lemon extract intake (Table 3). Remarkably, the excretion of hesperetin metabolites (**M7**, **M8**, **M14**) was higher (around 2-fold) after consuming lemon extract than orange extract (Table 4). This is especially relevant, considering that hesperidin consumption was around 13-fold higher after orange extract vs. lemon extract intake (Table 2).

The peak excretion of metabolites occurred in F2, from 4 to 8 h, after consuming lemon extract, being **M6** (homoeriodictyol glucuronide), the metabolite with the highest excretion (Table 4). In contrast, the metabolites excretion in F2 and F3 was very similar after the orange extract intake, being **M8** (hesperetin 3′-*O*-glucuronide) the primary metabolite excreted but showing 2.5-fold less excretion than after lemon consumption. The overall excretion of metabolites was substantially higher after consuming lemon than orange extract (339 ± 482 vs. 28 ± 55 µg/mg creatinine, respectively) (Table 4).

Interestingly, phase-II metabolites derived from eriodictyol (**M1**, **M2**, **M4**, and **M13**) were also quantified after orange extract intake. The glucuronide metabolites derived from eriodictyol represented 23% of the total, whereas hesperetin glucuronides accounted for 55% of the total. Hesperetin glucuronides peaked from 4 to 10 h, and eriodictyol glucuronides mainly peaked at 10–24 h.

It is interesting to note the large interindividual variability observed in the urinary excretion of metabolites. Supplementary Figure S2 illustrates the total excretion of the primary metabolites excreted **(M6**, **M8**, **M10**, and **M14**) for each volunteer after consuming the extracts. Homoeriodictyol glucuronide (**M6**) was not quantified in urine (Table 4), but it was detected in three volunteers after consuming orange extract (Table 3, Supplementary Figure S2). These volunteers (volunteers 2, 7, and 12) also excreted the highest concentrations of **M8** (hesperetin 3′-*O*-glucuronide) after consuming the orange extract.

None of the subjects had measurable concentrations of either hesperidin or eriocitrin-derived metabolites in plasma at baseline. All plasma metabolites, except **M11** (hesperetin sulfoglucuronide), were present in most volunteers after lemon extract intake, while fewer metabolites were detected after orange extract intake (Table 3; Figure 2). The highest plasma C_max_ values of phase-II metabolites were those derived from eriodictyol (**M1**, **M2**, **M4,** and **M13**), homoeriodictyol (**M6** and **M10**), and hesperetin (**M7**, **M8,** and **M14**) after consuming lemon extract (Table 5). Eriodictyol and homoeriodictyol-derived metabolites were also quantified in the plasma of some volunteers after orange extract intake, but only in some volunteers and at specific time-point blood withdrawals. Remarkably, C_max_ values of hesperetin metabolites **M7** (169.1 ± 109.4 nM), **M8** (198.5 ± 103.7 nM), and **M14** (527.0 ± 357.8 nM) were significantly higher after lemon than orange extract intake (49.6 ± 51.7, 106.8 ± 128.5, and 177.9 ± 285.8 nM, respectively) (Table 5), despite the higher amount of hesperidin ingested with the orange extract (Table 2).

Similar to urine samples, volunteers 2, 7, and 12 showed the highest concentrations for these metabolites as well as for phase-II hesperetin metabolites after lemon and orange extract intake, respectively (Supplementary Figure S3). Remarkably, the narigenin glucuronides **M3** and **M5** were also quantified in plasma after orange extract intake despite the extract lacked either naringin or naringenin. Similar to the urinary excretion, plasma metabolite concentrations also showed a high interindividual variability (Supplementary Figure S3).

The mean values of maximum concentrations times (T_max_) ranged from 5.4 to 6.5 h and 7.3 to 8.4 h after consuming lemon or orange extract, respectively. Overall, total plasma metabolites showed significantly mean lower T_max_ (6.0 ± 0.3 vs. 8.0 ± 0.5 h) and higher C_max_ (5.7 ± 5.4 vs. 0.35 ± 0.5 μM) and AUC values (21.1 ± 24.5 vs. 1.3 ± 2.1 μM h) after consuming lemon vs. orange extract, respectively (Table 5) (C_max_ and AUC values were significantly higher after consuming lemon vs. orange extract, taking into account the different amounts of flavanones consumed).

**Figure 3:**
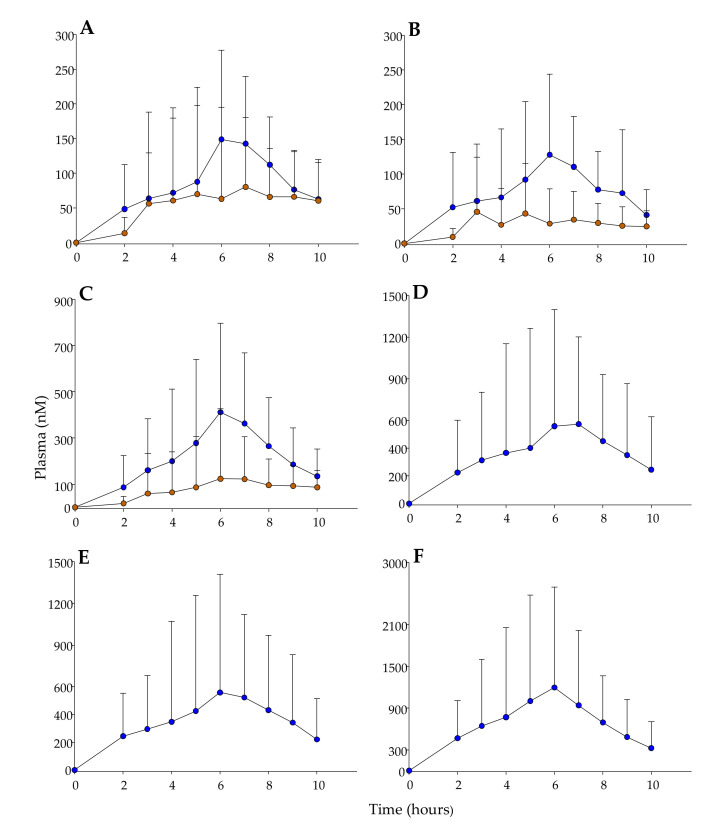
Plasma pharmacokinetic profiles of different metabolites after consuming lemon (blue) or orange (brown) extracts. (**A**) **M8** (hesperetin 3′-*O*-glucuronide); (**B**) **M7** (hesperetin 7-*O*-glucuronide); (**C**) **M14** (hesperetin 3′-*O*-sulfate); (**D**) **M2** (eriodictyol glucuronide isomer-2); (**E**) **M6** (homoeriodictyol glucuronide); and (**F**) **M13** (eriodictyol sulfate). Results are expressed as mean ± SD.

Figure 3 shows the pharmacokinetic profiles of representative metabolites after consuming lemon or orange extract. The previously mentioned high interindividual variability allowed us to stratify the volunteers into high and low metabolite producers. In this regard, Supplementary Figure S4 shows the plasma concentrations of **M2** and **M8** in a low producer (volunteer 6) vs. a high producer (volunteer 2). Remarkably, the higher plasma concentration detected after consuming lemon extract allowed the detection of both metabolites in both high- and low-producers, while **M2** was detected after consuming orange extract in the high producer, but not in the low producer (Supplementary Figure S4).

### 3.5. Human Metabolism of Eriocitrin and Hesperidin to Yield Phase-II Metabolites

**Figure 4:**
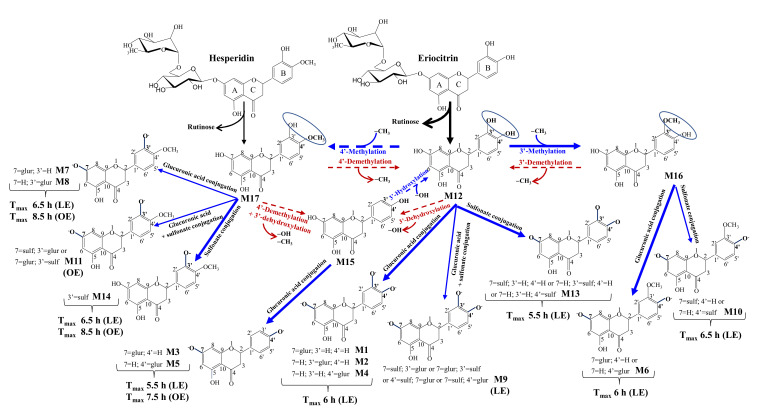
Main metabolic transformations of hesperidin and eriocitrin to their corresponding phase-II conjugates. The identification and quantification of metabolites (M) can be found in Table 3 and Table 4, respectively. The pharmacokinetic values can be found in Table 5. Black lines, microbial metabolism; blue lines, phase-II metabolism; red lines, phase-I or microbial metabolism; dashed lines, proposed new metabolic steps in the human metabolism of hesperidin and eriocitrin. The thickness of the lines represents the most favored pathways. LE, lemon extract; OE, orange extract (hesperidin); glur, glucuronic acid; sulf, sulfonic acid. The formation of eriodictyol (**M12**) from hesperetin mainly occurs via 4′-demethylation of hesperetin (**M17**) and the formation of naringenin (**M15**), after 4′-demethylation plus 3′-dehydroxylation. Besides, **M12** could also be formed after CYP-catalyzed 3′-hydroxylation of **M15** (Figure 4). These steps were supported by the detection of eriodictyol and naringenin conjugates after hesperidin intake in plasma and urine. However, no homoeriodictyol or derived metabolites (**M16**, **M6**, **M10**) were observed after hesperidin intake, which indicates that 4′-methylation to yield back hesperetin was favored instead of the subsequent 3′-methylation of eriodictyol to yield homoeriodictyol after consuming hesperidin (Figure 4).

Our results support the hypothesis that the higher solubility of eriocitrin vs. hesperidin would greatly favor the hydrolytic action of microbial rhamnosidases to catalyze the removal of the rutinose moiety to yield eriodictyol (**M12**). Once produced, **M12** was either: (i) methylated in the 3′- position by the enzyme catechol-*O*-methyl transferase (COMT) to yield homoeriodictyol (**M16**) and derived conjugates (**M6** and **M10**), or (ii) conjugated to yield mainly eriodictyol glucuronide (**M1**, **M2**, **M4**) or sulfate (**M13**) conjugates, or (iii) 3′-dehydroxylated to yield naringenin (**M15**) and its glucuronide derivatives (**M3**, **M5**). Remarkably, our results describe for the first time in humans the formation of hesperetin (**M17**) after consuming eriocitrin, likely due to the methylation of eriodictyol in the 4′ position by COMT (Figure 4).

## 4. Discussion

In the present study, we mainly aimed at evaluating whether the higher solubility of eriocitrin vs. hesperidin could enhance the metabolism of eriocitrin to yield phase-II derived metabolites in a shorter time, including eriodictyol-derived metabolites, with increased antioxidant activity. The volunteers consumed a single dose of 744 mg and 260 mg citrus flavanones after consuming lemon and orange extract, respectively, with equal administration, 260 mg of eriocitrin (lemon) and hesperidin (orange). We did not expect to find an “orange extract” with only hesperidin as a phenolic component since orange is a source also rich in other flavanones [6]. Moreover, we want to emphasize that we focused on the metabolism of eriocitrin and hesperidin to yield phase-II flavanone metabolites, the bottleneck in the bioavailability and metabolism of flavanones, and not comprehensively characterize the metabolic profile of microbial-derived ring fission phenolic catabolites [6], which will be addressed in future studies.

In general, the flavanone amounts administered in human interventions to evaluate hesperidin effects range from 146 to 1000 mg, with an average dose of ~500–600 mg [35]. Moreover, flavanone effects are usually evaluated for weeks or months [36,37]. Nevertheless, we also explored as a secondary outcome the post-prandial effects on different biochemical, metabolic, inflammatory, and oxidative markers after a high-fat-high-sugar meal.

In the present study, the meal was consumed 5.5 h after consuming the extracts. The hypothesis was that a representative pool of circulating phase-II flavanone metabolites would be present in the bloodstream to counteract the post-prandial deleterious effects of the high-fat-high-sugar meal potentially. Overall, no significant post-prandial changes were observed in the metabolic, oxidative stress, and inflammatory biomarkers upon consumption of the extracts, in agreement with Schär et al. [25], who did not observe significant differences on several cardiovascular risk markers between baseline and 5 h post-intake of orange juice containing 320 mg hesperidin.

The background that reports beneficial effects after hesperidin or hesperidin-rich foods intake is mostly supported by long-term interventions, weeks or even months, identifying hesperetin metabolites as key bioactive compounds [5,38]. A recent study in semi-professional cyclists showed that an acute and single dose of 500 mg of hesperidin improved physical performance, modulating oxidative status in the volunteers [39]. Nevertheless, the vast majority are long-term studies focused on cardiovascular risk factors, and their results are sometimes contradictory [40,41]. Whereas a recent study showed a reduction of glucose, insulin, triglycerides, cholesterol, LDL-c, and HOMA-IR index after two months of orange juice consumption in females [41], another study did not observe changes in these markers after a daily dose of 800 mg of hesperidin [40]. On the other hand, we found only one trial that evaluated the effects of eriocitrin in prediabetic individuals [35] and reported an improvement of hyperglycemic, inflammatory, and metabolic parameters, but not dose-dependently after consuming 200–800 mg/day eriocitrin for three months [35]. These results suggest that consistent consumption of lower eriocitrin doses could exert beneficial health effects.

In our present study, from the oxylipin-related HODEs metabolites, markers of oxidative stress, and components of the atherosclerotic plaque, only 9,12,13-(TriHOME), 12(13)-DiHOME, and 9(10)-DiHOME increased after the high-fat-high-sugar meal in both groups. This result may indicate a potential protective effect of both citrus extracts’ consumption since other HODE derivatives, such as 9-HODE and 13-HODE, have been reported to increase in humans after a high-fat meal [42].

The high-fat, high-sugar meal, unexpectedly, did not significantly increase glucose values over the baseline, which could support the hypoglycemic effects described upon citrus intake [43,44]. However, the meal significantly increased triglycerides, insulin, HOMA-IR, and heart rate, independently of the citrus extract consumed. Remarkably, systolic blood pressure increased slightly, but significantly, after the meal when volunteers ingested the orange extract, but not after consuming the lemon extract. Although the antioxidant and anti-inflammatory activities of flavanones have been widely acknowledged, no studies have compared their potential effects in humans so far. We did not find relevant post-prandial effects of the citrus extracts after a high-fat-high-sugar meal, which supports that the design to explore short-term and acute effects might not be appropriate for flavanones. Nevertheless, an improvement of the antioxidant status cannot be discarded through the modulation of other targets, such as the Nrf2 signaling pathway as previously reported [45]. Therefore, considering the significant role of the gut microbiota in the flavanones-related effects, evaluating the long-term health effects of citrus flavanones seems to be a better scenario. In this regard, the health effects of a lemon aqueous-soluble extract upon chronic consumption should be investigated.

Regarding the primary endpoint of the present study, the eriocitrin-rich lemon extract intake provided higher circulating plasma concentrations of phase-II conjugated flavanones than hesperidin. Although we are aware of the limitations of comparing a lemon extract with an orange extract that was actually hesperidin, we have focused on comparing eriocitrin vs. hesperidin metabolisms. In this regard, a remarkable (around 11–fold) increase of hesperetin-derived metabolites was observed after lemon vs. orange extract intake, considering the different amount of hesperidin in both extracts as well as other possible hesperetin precursors, including diosmetin and limocitrin derivatives in the lemon extract (Table 2). Our results suggest that lemon extract could enhance hesperetin generation in the gut mainly via eriodictyol formation through eriocitrin intake. In this way, the low solubility of hesperidin that severely hampers its metabolism by microbial rhamnosidases could be overcome. Overall, COMT-catalyzed reactions are expected since COMT action aims at counteracting the high reactivity of catechol (*ortho*-dihydroxyl) groups, such as that found in the B-ring of eriodictyol (M12) (Figure 4) [46]. These results agree with a recent study that tentatively identified hesperetin and homoeriodictyol in male Sprague-Dawley rats after administrating 50 mg/kg eriocitrin by gavage [47]. Nevertheless, we acknowledge that consumption of other minor possible hesperetin precursors in the lemon extract could also contribute to hesperetin formation. For example, diosmetin derivatives can yield hesperetin upon reducing the 2,3 double bond of the C-ring, as previously described in one volunteer that consumed 1 g diosmin [48]. The tentative 8-demethylation of limocitrin-HMG-Glu could also yield hesperetin, although this has not been described so far (Figure 4).

Our study also shows the formation of eriodictyol and homoeriodictyol conjugates after hesperidin intake. Another recent study in rats highlighted the presence of these metabolites upon demethylation of hesperetin by phase-I enzymes [49]. Other authors have also identified eriodictyol metabolites [6] and quantified eriodictyol-sulfate and eriodictyol-*O*-glucuronyl-sulfate in humans after orange juice intake [50]. However, the orange juice consumed in these studies already contained eriocitrin. In our present study, hesperidin was the only flavanone found in the commercial orange extract, and thus, our results demonstrate for the first time the human production of eriodictyol and narigenin metabolites from hesperidin unequivocally. This process seems to occur via 4′-demethylation reactions likely catalyzed by phase-I enzymes [49], although the involvement of microbial demethylases cannot be ruled out. Of note, Matsumoto et al. [51] reported homoeriodictyol glucuronide in rat plasma after oral hesperidin administration, but these authors did not detect eriodictyol conjugates. Overall, these data suggest a two-way dynamic methylation-demethylation process between hesperetin and eriodictyol (Figure 4).

We have also identified naringenin metabolites (Figure 4) in most of the volunteers (Table 3, Table 4 and Table 5) after consuming both extracts. Unlike lemon extract, which contained naringenin, hesperidin consumption allowed us to confirm the transformation of hesperetin (M17) to naringenin (M15) unequivocally, most likely due to a 4′-demethylation plus 3′-dehydroxylation from M12 to M15 (Figure 4). This dihydroxylation process would agree with that observed in vivo from quercetin to kaempferol [52]. To the best of our knowledge, this is the first report that describes the formation of naringenin from hesperetin in humans. Increased production of narigenin-derived metabolites could play an essential role in preventing cardiovascular and metabolic diseases [53], although only a few human studies have been carried out to evaluate the effects of naringenin [54].

The high interindividual variability observed in the production of flavanone metabolites was not abolished after lemon extract intake. Although the gut microbial metabolism seemed to be favored, however, phase-I and phase-II enzymes also contribute to the high interindividual variability of polyphenols metabolism as previously suggested [17,46,55]. Nevertheless, lemon extract intake provided more bioavailable and a higher concentration of phase-II flavanones than hesperidin, which could be an opportunity to enhance the metabolite production of those low flavanone metabolite producers [18,56]. Therefore, lemon extract intake could provide a sufficient circulating concentration metabolites threshold to exert health benefits even in low producer individuals. Our results suggest that, unlike lemon extract, a commercial “orange extract” based on purified hesperidin does not seem to be a suitable alternative to other natural orange flavanone sources such as orange juice.

We acknowledge some limitations in our study. Although post-prandial vs. baseline changes were compared in each individual, we lacked a control group with no citrus extract consumption to draw unequivocal conclusions on the differences in metabolic, biochemical, and oxidative markers. Moreover, the exact comparison of the quantitative production of some flavanone metabolites was not possible since the orange extract only contained hesperidin, unlike the richer flavanone profile of the lemon extract. In this regard, it could have also been interesting to conduct a parallel study with pure compounds to evaluate how much the extract matrix influences these molecules’ pharmacokinetics. On the other hand, the intake of hesperidin allowed us to confirm some metabolic steps unequivocally. We have focused on eriocitrin and hesperidin metabolism and their phase-II flavanone metabolism. However, much more information remains to be elucidated regarding the microbial-derived ring fission phenolic catabolites upon comparing lemon extract vs. hesperidin metabolism.

## 5. Conclusions

Taking into account the results and our limitations, we have reported new insights into the human metabolism of eriocitrin and hesperidin. Our findings support that eriocitrin rich-lemon extract intake provides more circulating phase-II flavanone metabolites and higher concentrations than after consuming hesperidin-rich extracts. Our results also suggest that lemon extract intake could provide a sufficient circulating concentration metabolites threshold to exert health benefits upon long-term consumption, even in the so-called low-producer individuals. This could be relevant to overcome the disparate flavanones-related health-effects observed in human subjects. The investigation of health-related effects after regular consumption of this aqueous-soluble and eco-friendly eriocitrin-rich extract is warranted.

## Figures and Tables

**Table 1:** Subjects characteristics and baseline values of the volunteers ^1^.

		Baseline LE ^2^	Baseline OE ^2^
**Subjects characteristics**			
Age (years)	33.2 ± 7.2 (26–49)		
Weight (kg)	68.1 ± 13.2 (48–90)		
BMI (kg/m^2^)	23.0 ± 2.1 (19–25)		
Sex (Female/Male)	8/8		
**Baseline values ^1^**			
Glucose (mg/dL)		81.8 ± 8.3 (67–98)	79.4 ± 7.5 (69–96)
Total cholesterol (mg/dL)		169.7 ± 30.2 (129–238)	164.7 ± 24.9 (121–217)
HDL-cholesterol (mg/dL)		56.7 ± 12.6 (33–89)	53.4 ± 11.8 (29–81)
LDL-cholesterol (mg/dL)		97.1 ± 25.3 (64–157)	93.6 ± 21.2 (62–140)
Triglycerides (mg/dL)		72.6 ± 43.2 (34–173)	74.7 ± 39.0 (35–150)
Insulin (µU/mL)		4.0 ± 1.9 (2–7)	3.5 ± 2.2 (1–9)
HOMA-IR		0.8 ± 0.4 (0.3–1.4)	0.7 ± 0.5 (0.2–2.0)
GGT (U/mL)		15.3 ± 9.1 (6–36)	15.3 ± 7.9 (8–34)
ALT (U/mL)		19.5 ± 8.7 (10–39)	19.9 ± 9.4 (11–41)
Diastolic blood pressure (mmHg)		71.5 ± 8.7 (58–91)	71.3 ± 10.8 (57–90)
Systolic blood pressure (mmHg)		110.4 ± 10.8 (96–129)	109.9 ± 11.0 (90–127)
Heart rate (bpm)		59.4 ± 8.9 (42–76)	55.5 ± 7.4 (43–68)

^1^ Values are shown as mean ± SD (range) (*n* = 16). ^2^ Mean baseline data from 16 volunteers before the administration of the two citrus extracts (*n* = 32); HOMA-IR, homeostatic model assessment for insulin resistance; GGT, gamma-glutamyl transferase; ALT, alanine aminotransferase; LE, lemon extract; OE, orange extract.

**Table 2:** Phenolic compounds identified in the citrus extracts by HPLC-DAD-MS/MS.

Peak Nº	Compound	RT	*m*/*z*^-^	MS/MS	λ_max_	mg/g Extract ^1^	Extract
1	Ferulic acid-*O*-Glu	16.94	355	193/160/134	294/328	10.40 ± 0.51	Lemon
2	Apigenin 6,8-di-*C*-Glu (Vicenin-2)	18.13	593	503/473/383/353	270/332	14.6 ± 0.4	Lemon
3	Eriodictyol-Glu-Rha-Glu	18.70	757	595/449/287	282/330	33.86 ± 0.91	Lemon
4	Diosmetin 6,8-di-*C*-Glu	19.00	623	605/533/503/383	270/346	9.62 ± 0.27	Lemon
5	Crisoeriol 6,8-di-*C*-Glu	19.65	623	533/503/383	270/346	11.3 ± 0.2	Lemon
6	Eriocitrin	22.90	595	459/329/287	284/334	83.3 ± 5.6	Lemon
7	Apigenin 8-*C*-xylanopyranosil-Glu	23.39	563	413/341/293	268/332	10.0 ± 0.15	Lemon
8	Diosmetin 8-*C*-Glu	24.88	461	371/341	268/334	14.31 ± 0.35	Lemon
9	Naringenin 7-*O*-rutinoside (naringin)	25.02	579	271	278/330	35.45 ± 6.04	Lemon
10	Hesperidin	28.10	609	301	284/336	6.3 ± 0.1 133.7 ± 15.9	Lemon *Orange*
11	Diosmetin 7-*O*-rutinoside (Diosmin)	28.40	607	299/284	270/334	4.12 ± 0.13	Lemon
12	Limocitrin-HMG-Glu	29.43	651	549/507/345	274/350	6.9 ± 0.2	Lemon
**Total:**						**240.16 ± 14.86**	**Lemon**
						**133.7 ± 15.9**	***Orange***

^1^ Values are shown as mean ± SD (*n* = 3). Nº, peak number in Figure S1; RT, retention time; Glu, glucoside; Rhamn, rhamnoside; HMG, 3-hydroxy-3-methyl-glutaryl-.

**Table 3:** Plasma and urine metabolites identified in volunteers (*n* = 16) after consuming lemon (LE) or orange (OE) extracts.

N^º^	Metabolites	RT	*m*/*z*^-^	Occurrence ^2^			
				Urine LE	Plasma LE	Urine OE	Plasma OE
**M1**	Eriodictyol glucuronide-1	6.37	463.0882	16	16	5	3
**M2**	Eriodictyol glucuronide-2	7.31	463.0882	16	16	10	7
**M3**	Naringenin 7-*O*-glucuronide ^1^	7.52	447.0933	16	16	16	14
**M4**	Eriodictyol glucuronide-3	7.68	463.0882	16	16	11	7
**M5**	Naringenin 4′-*O*-glucuronide ^1^	7.70	447.0933	16	16	16	14
**M6**	Homoeriodictyol glucuronide	7.72	477.1038	16	16	3	3
**M7**	Hesperetin 7-*O*-glucuronide ^1^	8.02	477.1038	16	16	16	16
**M8**	Hesperetin 3′-*O*-glucuronide ^1^	8.20	477.1038	16	16	16	16
**M9**	Eriodictyol sulfoglucuronide	8.99	543.0450	5	15	0	0
**M10**	Homoeriodictyol sulfate	9.02	381.0286	16	16	0	3
**M11**	Hesperetin sulfoglucuronide	9.30	557.0607	0	0	7	8
**M12**	Eriodictyol ^1^	9.50	287.0561	14	16	4	0
**M13**	Eriodictyol sulfate	9.75	367.0129	16	16	12	10
**M14**	Hesperetin 3′-*O*-sulfate ^1^	9.89	381.0286	16	16	16	16
**M15**	Naringenin ^1^	11.22	271.0612	9	6	5	2
**M16**	Homoeriodictyol ^1^	11.42	301.0718	12	15	1	1
**M17**	Hesperetin ^1^	11.73	301.0718	5	8	8	3

^1^ The identification was achieved using authentic standards. ^2^ Number of volunteers in whom each metabolite was detected after consuming lemon (LE) or orange (OE) extracts.

**Table 4:** Metabolites quantified in urine fractions (Figure 1) after consuming lemon or orange extract.

		Lemon Extract					Orange Extract				
Nº	Metabolites	F1	F2	F3	F4	Total F	F1	F2	F3	F4	Total F
**M1**	Eriodictyol glucuronide-1	3.2 ± 5.9	9.6 ± 15.5	4.5 ± 6.2	0.7 ± 0.7	18.0 ± 28.4	–	0.4 ± 0.3	0.1 ± 0.1	0.05 ± 0.05	0.6 ± 0.4
**M2**	Eriodictyol glucuronide-2	6.4 ± 11.0	18.0 ± 17.2	18.1 ± 19.7	4.2 ± 5.7	46.6 ± 53.6 **	0.1 ± 0.1	0.5 ± 0.8	0.5 ± 0.6	1.4 ± 3.4	2.5 ± 4.9
**M3**	Naringenin 7-*O*-glucuronide	1.0 ± 0.7	2.6 ± 4.0	1.7 ± 1.9	0.3 ± 0.3	5.7 ± 6.9 ***	0.1 ± 0.1	0.2 ± 0.6	0.2 ± 0.2	0.2 ± 0.3	0.7 ± 1.5
**M4**	Eriodictyol glucuronide-3	9.4 ± 16.9	33.8 ± 64.1	27.7 ± 40.1	4.3 ± 6.4	75.1 ± 128 *	0.1 ± 0.2	0.5 ± 0.9	0.7 ± 0.7	2.1 ± 4.5	3.4 ± 6.2
**M5**	Naringenin 4′-*O*-glucuronide	0.8 ± 0.5	1.7 ± 2.7	1.3 ± 1.5	0.4 ± 0.3	4.2 ± 5.0 ***	0.1 ± 0.1	0.3 ± 0.5	0.3 ± 0.4	0.3 ± 0.5	1.0 ± 1.5
**M6**	Homoeriodictyol glucuronide	13.9 ± 16.2	48.1 ± 90.0	37.2 ± 56.9	6.8 ± 8.0	106 ± 171	–	–	–	–	–
**M7**	Hesperetin 7-*O*-glucuronide	1.2 ± 1.4	4.0 ± 4.7	2.7 ± 2.5	0.6 ± 0.6	8.5 ± 9.2 ***	0.2 ± 0.6	2.1 ± 5.7	2.2 ± 3.6	1.1 ± 3.3	5.6 ± 13.2
**M8**	Hesperetin 3′-*O*-glucuronide	2.1 ± 2.3	10.7 ± 11.3	9.2 ± 7.7	2.2 ± 2.4	24.2 ± 23.7 ***	0.5 ± 1.5	4.3 ± 11.5	3.3 ± 4.2	1.8 ± 1.6	9.9 ± 18.9
**M10**	Homoeriodictyol sulfate	3.8 ± 3.2	4.0 ± 5.5	3.6 ± 4.1	0.5 ± 0.4	9.6 ± 11.3	–	–	–	–	–
**M13**	Eriodictyol sulfate	4.6 ± 5.0	14.6 ± 17.3	11.9 ± 12.6	1.5 ± 1.5	32.6 ± 36.5 ***	0.02 ± 0.01	0.1 ± 0.1	0.2 ± 0.4	0.3 ± 0.4	0.6 ± 0.9
**M14**	Hesperetin 3′-*O*-sulfate	1.9 ± 2.6	4.3 ± 4.5	3.3 ± 3.1	0.7 ± 0.6	9.0 ± 9.1 *	0.1 ± 0.3	1.2 ± 3.3	1.9 ± 3.1	0.6 ± 0.6	3.8 ± 7.3
**Total excretion (24 h):**						339 ± 482 ***					**28 ± 55**

Values expressed as µg/mg creatinine (mean ± SD). Nº, metabolite according to Figure 2 and Table 3. F1: Fraction collected from 0 to 4 h after consuming lemon or orange extract; F2: Fraction collected from 4 to 8 h; F3: Fraction collected from 8 to 10 h; F4: Fraction collected at 24 h; Total F (24 h), sum of the fractions F1, F2, F3 and F4. * *p* < 0.05; ** *p* < 0.01; *** *p* < 0.001.

**Table 5:** Plasma pharmacokinetic parameters of the quantified metabolites after consuming lemon or orange (hesperidin) extract.

		T_max_ (h)		C_max_ (nM)		AUC_0-t_ (nM·h)	
Nº	Metabolites	Lemon	Hesperidin	Lemon	Hesperidin	Lemon	Hesperidin
**M1**	Eriodictyol glucuronide-1	5.9 ± 2.4	—	103.9 ± 85.8	—	282.6 ± 271.3	—
**M2**	Eriodictyol glucuronide-2	5.9 ± 2.4	—	871.3 ± 868.0	—	3222 ± 4118	—
**M3**	Naringenin 7-*O*-glucuronide	5.4 ± 2.5 *	7.3 ± 2.2	56.7 ± 44.2 ***	4.7 ± 3.6	202.7 ± 187.1 ***	18.8 ± 19.0
**M4**	Eriodictyol glucuronide-3	5.9 ± 2.6	—	638.6 ± 801.8	—	2291 ± 3491	—
**M5**	Naringenin 4′-*O*-glucuronide	5.7 ± 2.3 *	7.7 ± 2.0	35.8 ± 25.9 ***	7.3 ± 5.9	129.4 ± 124.0 ***	26.7 ± 27.7
**M6**	Homoeriodictyol glucuronide	6.1 ± 2.4	—	780.5 ± 863.2	—	3133 ± 4170	—
**M7**	Hesperetin 7-*O*-glucuronide	6.3 ± 2.0 *	8.3 ± 1.6	169.1 ± 109.4 ***	49.6 ± 51.7	703.3 ± 590.5 ***	176.7 ± 249.5
**M8**	Hesperetin 3′-*O*-glucuronide	6.4 ± 2.0 *	8.4 ± 1.6	198.5 ± 103.7 *	106.8 ± 128.5	802.8 ± 568.8 *	408.9 ± 643.2
**M10**	Homoeriodictyol sulfate	6.5 ± 2.2	—	479.4 ± 524.0	—	1778 ± 2257	—
**M13**	Eriodictyol sulfate	5.7 ± 2.5	—	1706 ± 1510	—	6120 ± 6631	—
**M14**	Hesperetin 3′-*O*-sulfate	6.5 ± 1.9 ***	8.4 ± 1.5	527.0 ± 357.8 ***	177.9 ± 285.8	2037 ± 1809 ***	635.3 ± 1167
	**Total:**			5724 ± 5432 *	**347 ± 475**	21060 ± 24531 *	**1266 ± 2106**

**Nº**, metabolite according to Figure 2 and Table 3; –, not determined; T_max_, time of maximum concentration; C_max_, maximum concentration; AUC_0-t_, area under the curve from the time of dosing to the final quantifiable concentration. Asterisks designate a significant difference between consumption of lemon extract and hesperidin (* *p* < 0.05; *** *p* < 0.001).

## Data Availability

Fully anonymized data presented in this study can potentially be available upon specific request at the discretion of the corresponding author. The data are not publicly available for privacy and ethical reasons given that the research participants who consented for the study did not provide specific consent to have their data shared in a public database.

## Supplementary Materials

The following are available online at https://www.mdpi.com/2076-3921/10/3/435/s1, Table S1. Markers associated with inflammation and oxidative stress; Figure S1. Chromatograms of lemon and orange extracts at 340 nm using HPLC-DAD-MS/MS; Table S2. Differences between postprandial and baseline values after consuming a high-fat-high-sugar meal and citrus extracts; Table S3. Oxylipins tentatively identified in plasma using UPLC-ESI-QTOF-MS; Table S4. Peak integration area values from the EICs of the identified plasma oxylipins; Figure S2. Total metabolite excretion (sum of urine fractions F1, F2, F3 and F4) of **M8**, **M14**, **M6** and **M10** after consuming lemon or orange extracts; Figure S3. Maximum plasma concentration (C_max_) of **M8**, **M14**, **M2**, and **M13** after lemon or orange extracts intake; Figure S4. Interindividual differences between low and high metabolite producers in the pharmacokinetics of **M8** and **M2**.
