# Supplementary material for: New Insights into the Metabolism of the Flavanones Eriocitrin and Hesperidin: A Comparative Human Pharmacokinetic Study
Source: Antioxidants (Basel). 2021 Mar 11;10(3):435. doi: 10.3390/antiox10030435 (PMC8000041; doi:10.3390/antiox10030435)
Supplement: Supplementary file 1 [file antioxidants-10-00435-s001.pdf]

SUPPLEMENTARY MATERIAL

New Insights into the Metabolism of the Flavanones Eriocitrin and Hesperidin: A Comparative Human Pharmacokinetic Study

*María Ángeles Ávila-Gálvez, Juan Antonio Giménez-Bastida, Antonio González-Sarrías and Juan Carlos Espín**

Laboratory of Food & Health, Research Group on Quality, Safety and Bioactivity of Plant Foods, CEBAS-CSIC, 30100 Campus de Espinardo, Murcia, Spain.

Correspondence:

*Address correspondence to J.C. Espín, Laboratory of Food & Health; Research Group on Quality, Safety and Bioactivity of Plant Foods; CEBAS-CSIC, 30100 Campus de Espinardo, Murcia, Spain. E-mail: jcespin@cebas.csic.es; Phone: +34-968-396200, Ext: 6344

Table S1. Markers associated with inflammation and oxidative stress.

Compounds	Abbreviation	Molecular formula	*m/z*⁻
8-Oxo-2'-deoxyguanosine	8-OHdG	$C_{10}H_{13}N_5O_5$	282.0844
13-Oxo-9,11-octadecadienoic acid	13-oxo-ODE	$C_{18}H_{30}O_3$	293.2100
13-Hydroxy-9,11-octadecadienoic acid	13-HODE	$C_{18}H_{32}O_3$	295.2279
9-Hydroxy-10,12-octadecadienoic acid	9-HODE	$C_{18}H_{32}O_3$	295.2279
12,13-Dihydroxy-9Z-octadecenoic acid	12(13)-DiHOME	$C_{18}H_{34}O_4$	313.2400
9,10-Dihydroxy-12Z-octadecenoic acid	9(10)-DiHOME	$C_{18}H_{34}O_4$	313.2400
12-Hydroxyeicosapentaenoic acid	12-HEPE	$C_{20}H_{30}O_3$	317.2122
15-Oxo-Eicosatetraenoic acid	15-oxo-ETE	$C_{20}H_{30}O_3$	317.2122
12-Oxo-Eicosatetraenoic acid	12-oxo-ETE	$C_{20}H_{30}O_3$	317.2122
20-Hydroxyeicosatetraenoic acid	20-HETE	$C_{20}H_{32}O_3$	319.2279
15-Hydroxyeicosatetraenoic acid	15-HETE	$C_{20}H_{32}O_3$	319.2279
12-Hydroxyeicosatetraenoic acid	12-HETE	$C_{20}H_{32}O_3$	319.2279
11-Hydroxyeicosatetraenoic acid	11-HETE	$C_{20}H_{32}O_3$	319.2279
9-Hydroxyeicosatetraenoic acid	9-HETE	$C_{20}H_{32}O_3$	319.2279
8-Hydroxyeicosatetraenoic acid	8-HETE	$C_{20}H_{32}O_3$	319.2279
5-Hydroxyeicosatetraenoic acid	5-HETE	$C_{20}H_{32}O_3$	319.2279
15-Hydroxy-8,11,13-eicosatrienoic acid	15-HETrE	$C_{20}H_{34}O_3$	321.2435
Prostaglandin M	PGM	$C_{16}H_{24}O_7$	327.1449
9,12,13-Trihydroxy-10-octadecenoic acid	9,12,13-(TriHOME)	$C_{18}H_{34}O_5$	329.2333
9,10,13-Trihydroxy-11-octadecenoic acid	9,10,13-(TriHOME)	$C_{18}H_{34}O_5$	329.2333
Leukotrien B4	LTB_4	$C_{20}H_{32}O_4$	335.2228
14,15-Dihydroxyeicosatrienoic Acid	14,15-DHET	$C_{20}H_{34}O_4$	337.2400
11,12-Dihydroxyeicosatrienoic Acid	11,12-DHET	$C_{20}H_{34}O_4$	337.2400
8,9-Dihydroxyeicosatrienoic Acid	8,9-DHET	$C_{20}H_{34}O_4$	337.2400
5,6-Dihydroxyeicosatrienoic Acid	5,6-DHET	$C_{20}H_{34}O_4$	337.2400
17-Hydroxydocosahexaenoic acid	17-HDoHE	$C_{22}H_{32}O_3$	343.2300
15-Keto-prostaglandin E2	15-keto-PGE2	$C_{20}H_{30}O_5$	349.2020
Prostaglandin E2	PGE2	$C_{20}H_{32}O_5$	351.2177
Prostaglandin D2	PGD2	$C_{20}H_{32}O_5$	351.2177
8-Iso-prostaglandin F2α	8-iso-$PGF_{2\alpha}$	$C_{20}H_{34}O_5$	353.2333
Prostaglandin E2-D4	PGE2-d4	$C_{20}H_{28}D_4O_5$	355.2428
Thromboxane B2	TXB_2	$C_{20}H_{34}O_6$	369.2283
Thromboxane B2-D4	TXB_2-d4	$C_{20}H_{30}D_4O_6$	373.2534
Resolvin D2	Rv-D2	$C_{22}H_{32}O_5$	375.2177
Resolvin D1	Rv-D1	$C_{22}H_{32}O_5$	375.2177
Hemiketal E2	HKE2	$C_{20}H_{32}O_8$	399.2024
Hemiketal D2	HKD2	$C_{20}H_{32}O_8$	399.2024
Leukotriene C4	LTC4	$C_{30}H_{47}N_3O_9S$	624.296

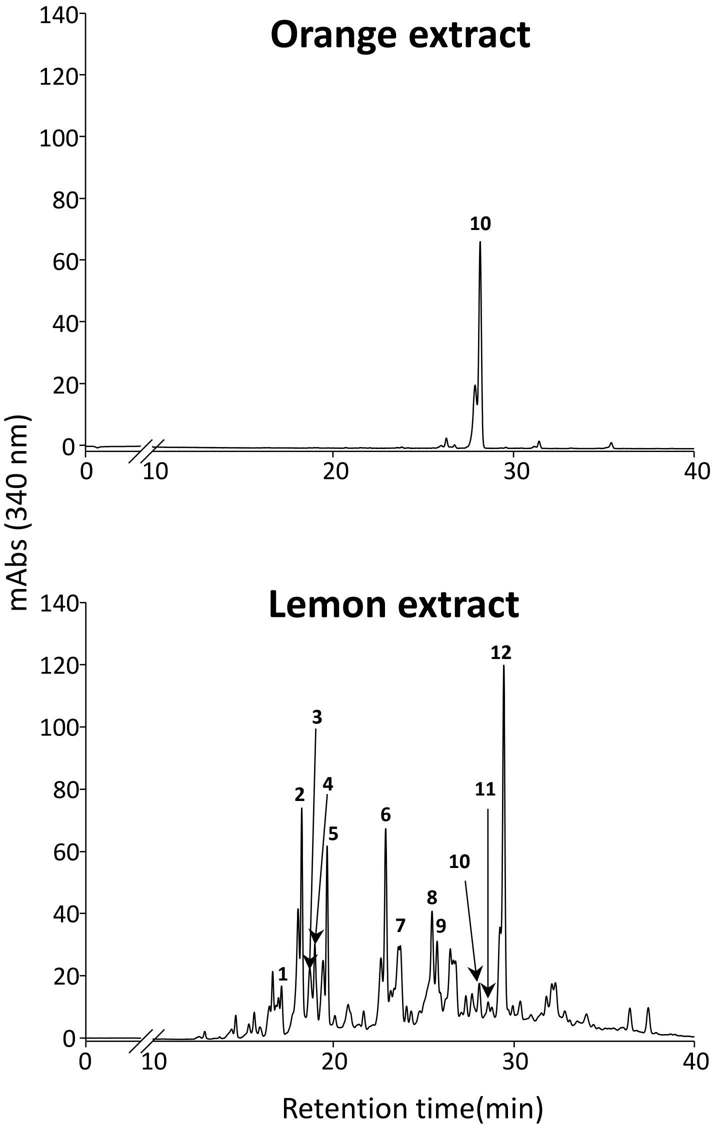

Figure S1. Chromatograms of lemon and orange extracts at 340 nm using HPLC-UV-MS/MS (IT). The numbers designate the compounds according to Table 2.

Table S2. Differences between postprandial and baseline values after consuming a high-fat-high-sugar meal and citrus extracts.

	Citrus extracts consumption			
	**Lemon**	***P***	**Orange**	***P***
	**Change (%)** (post-baseline)		**Change (%)** (post-baseline)	
Glucose (mg/dL)	15.0	0.071	9.3	0.202
Total cholesterol (mg/dL)	-1.2%	0.220	0.9	0.391
HDL-cholesterol (mg/dL)	0.9	0.770	0	1.000
LDL-cholesterol (mg/dL)	-7.4*	0.005	-4.9*	0.004
Triglycerides (mg/dL)	36.0*	< 0.001	40.8*	< 0.001
Insulin (μU/mL)	833*	< 0.001	1,237*	< 0.001
HOMA-IR	1,036*	< 0.001	1,414*	< 0.001
GGT (U/mL)	-1.3	0.486	-0.7	0.873
ALT (U/mL)	0	1.000	0.5	0.849
Diastolic blood pressure (mmHg)	0.6	0.637	-1.7	0.243
Systolic blood pressure (mmHg)	2.0	0.135	4.6*	0.026
Heart rate (bpm)	21.2*	< 0.001	22.5*	< 0.001

Values are shown as the difference (%) between postprandial (post) and baseline values after consuming a high-fat-high-sugar meal. *Significant difference. HOMA-IR, homeostatic model assessment for insulin resistance; GGT, gamma-glutamyl transferase; ALT, alanine aminotransferase.

Table S3. Oxylipins tentatively identified in plasma using UPLC-ESI-QTOF-MS.

Compounds	TR	*m/z*⁻ experimental	Molecular formula	Error (ppm)	Score
9,12,13-(TriHOME)	11,89	329.2331	$C_{18}H_{34}O_5$	0.73	99.12
9,10,13-(TriHOME)	13,15	329.2331	$C_{18}H_{34}O_5$	0.85	99.33
12(13)-DiHOME	16,14	313.2390	$C_{18}H_{34}O_4$	-1.50	98.70
9(10)-DiHOME	16,39	313.2383	$C_{18}H_{34}O_4$	0.49	99.04
13-oxo-ODE	17,91	293.2117	$C_{18}H_{30}O_3$	0.72	95.33
13-HODE	17,98	295.2278	$C_{18}H_{32}O_3$	-0.24	98.26
9-HODE	18,20	295.2274	$C_{18}H_{32}O_3$	1.49	98.08

Table S4. Peak integration area values from the EICs of the identified plasma oxylipins.

Compounds	LE-baseline	LE-post (1 h)	OE-baseline	OE-post (1 h)
9,12,13-(TriHOME)	(215±125) x 10^3	(753±1,006) x·10^{3}*	(221±113) x·10^3	(557±344) x·10^{3}***
9,10,13-(TriHOME)	(83±127) x·10^3	(64±197) x·10^3	(64±36) x·10^3	(110±127) x·10^3
12(13)-DiHOME	(959±435) x·10^3	(1,334±797) x·10^{3}*	(1,080±809) x·10^3	(1,295±893) x·10^{3}*
9(10)-DiHOME	(814±328) x·10^3	(1,209±515) x·10^{3}*	(937±818) x·10^3	(1,222±704) x·10^{3}*
13-oxo-ODE	(705±358) x·10^3	(964±691) x·10^3	(640±209) x·10^3	(782±319) x·10^3
13-HODE	(83±199) x·10^3	(87±203) x·10^3	(113±202) x·10^3	(138±250) x·10^3
9-HODE	(393±156) x·10^3	(453±269) x·10^3	(374±178) x·10^3	(357±139) x·10^3

Values are shown as mean±SD. *Significant differences between postprandial (1-hour post meal) and baseline values, after consuming a high-fat-high-sugar meal (*P<0.05; ***P<0.001); LE, lemon extract; OE, orange extract.

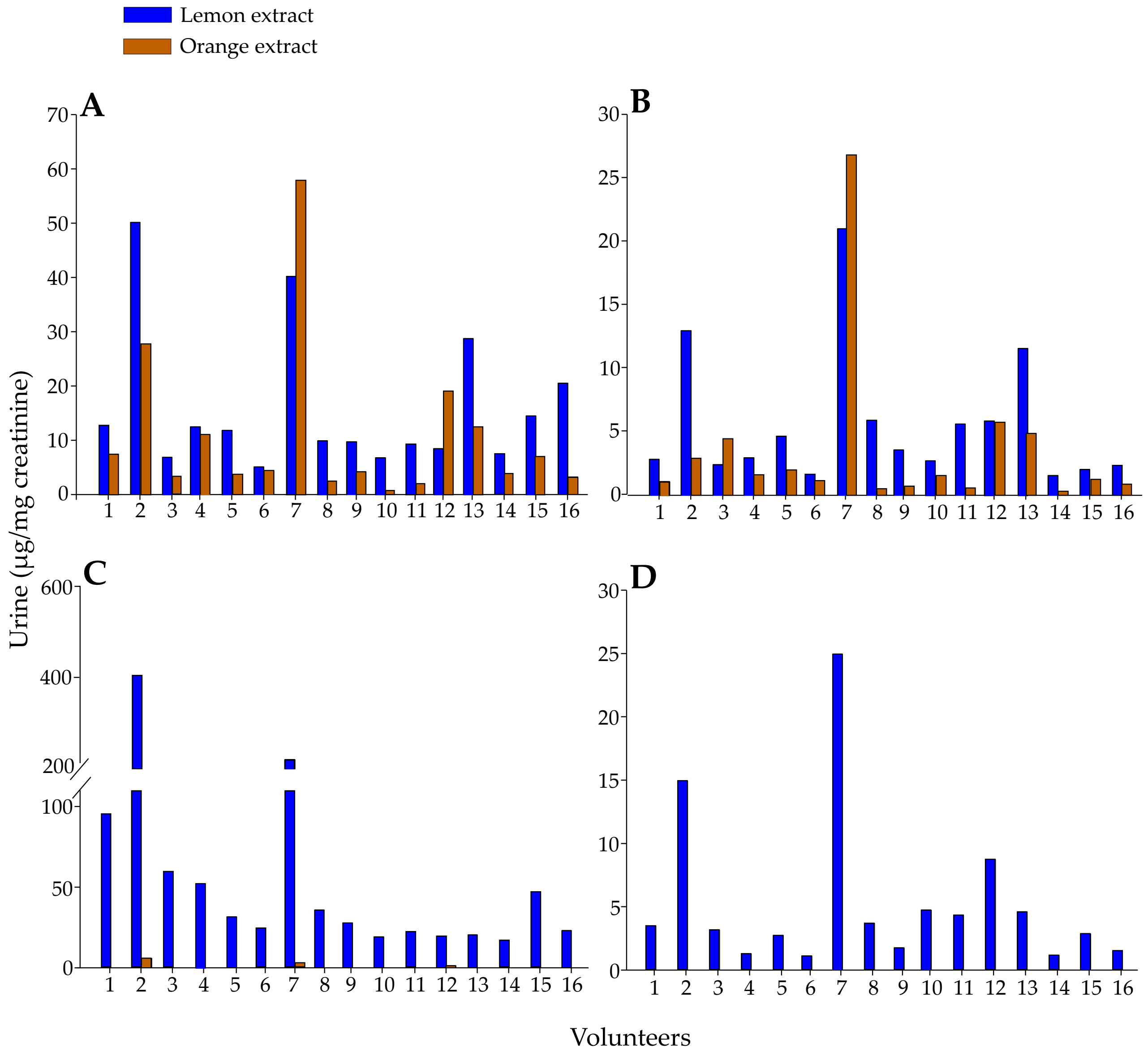

Figure S2. Total metabolite excretion (sum of urine fractions F1, F2, F3 and F4) of **M8**, **M14**, **M6** and **M10** after consuming lemon or orange extracts. (**A**) **M8** (hesperetin 3′-*O*-glucuronide); (**B**) **M14** (hesperetin 3′-*O*-sulfate); (**C**) **M6** (homoeriodictyol glucuronide); and (**D**) **M10** (homoeriodictyol sulfate). Blue bars, lemon extract; brown bars, orange extract (hesperidin).

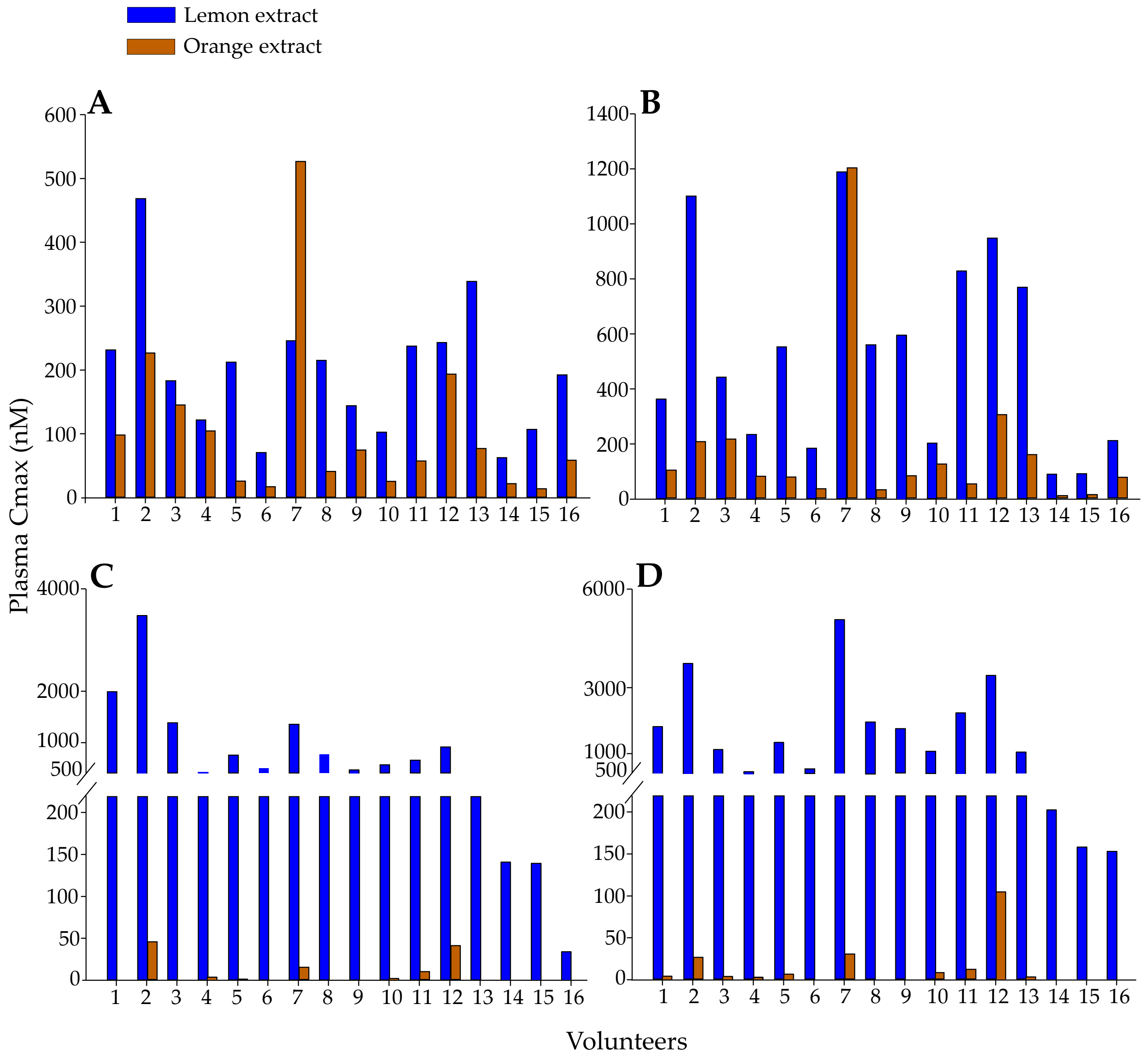

Figure S3. Maximum plasma concentration (C_{max}) of **M8**, **M14**, **M2**, and **M13** after lemon or orange extracts intake. (**A**) C_{max} values of **M8** (hesperetin 3′-*O*-glucuronide); (**B**) C_{max} values of **M14** (hesperetin 3′-*O*-sulfate); (**C**) C_{max} values of **M2** (eriodictyol glucuronide-2); and (**D**) C_{max} values of **M13** (eriodictyol sulfate). Blue bars, lemon extract; brown bars, orange extract (hesperidin).

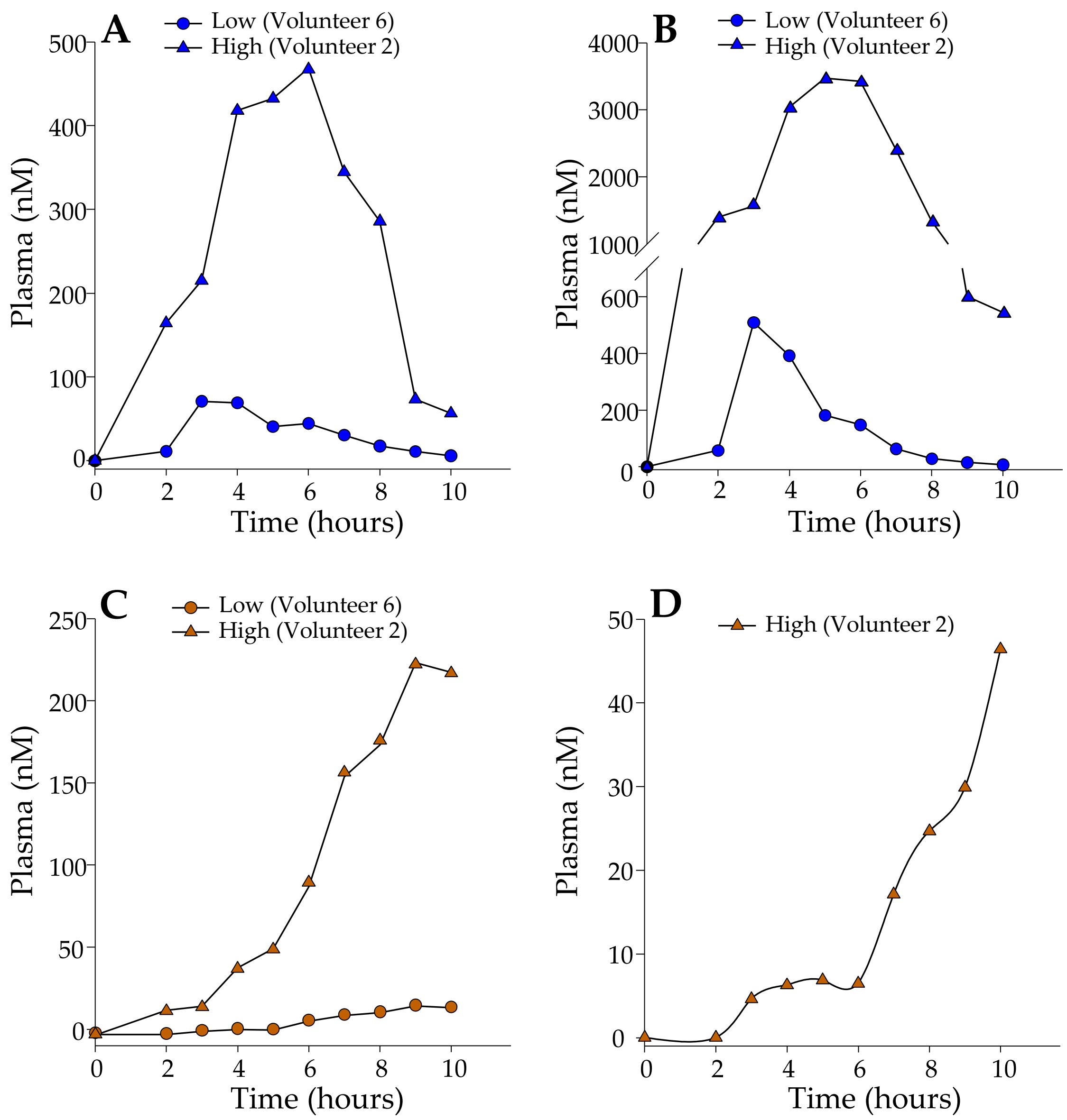

Figure S4. Interindividual differences between low and high metabolite producers in the pharmacokinetics of **M8** and **M2**. (**A**, **C**) **M8** (hesperetin 3′-*O*-glucuronide), and (**B**, **D**) **M2** (eriodictyol glucuronide-2), after consuming lemon (blue) or orange (brown) extracts. Circle, low producer; triangle, high producer.
